# T Cell Responses to Viral Infections – Opportunities for Peptide Vaccination

**DOI:** 10.3389/fimmu.2014.00171

**Published:** 2014-04-16

**Authors:** Sietske Rosendahl Huber, Josine van Beek, Jørgen de Jonge, Willem Luytjes, Debbie van Baarle

**Affiliations:** ^1^Centre for Infectious Disease Control, National Institute for Public Health and the Environment (RIVM), Bilthoven, Netherlands

**Keywords:** DC, peptides, vaccination, virus, infection, chronic, acute

## Abstract

An effective immune response against viral infections depends on the activation of cytotoxic T cells that can clear infection by killing virus-infected cells. Proper activation of these T cells depends on professional antigen-presenting cells, such as dendritic cells (DCs). In this review, we will discuss the potential of peptide-based vaccines for prevention and treatment of viral diseases. We will describe features of an effective response against both acute and chronic infections, such as an appropriate magnitude, breadth, and quality and discuss requirements for inducing such an effective antiviral immune response. We will address modifications that affect presentation of vaccine components by DCs, including choice of antigen, adjuvants, and formulation. Furthermore, we will describe differences in design between preventive and therapeutic peptide-based vaccines. The ultimate goal in the design of preventive vaccines is to develop a universal vaccine that cross-protects against multiple strains of the virus. For therapeutic vaccines, cross-protection is of less importance, but enhancing existing T cell responses is essential. Although peptide vaccination is successful in inducing responses in human papillomavirus (HPV) infected patients, there are still several challenges such as choosing the right target epitopes, choosing safe adjuvants that improve immunogenicity of these epitopes, and steering the immune response in the desired direction. We will conclude with an overview of the current status of peptide vaccination, hurdles to overcome, and prospects for the future.

## Introduction

Viruses are small infectious agents that consist of nucleic acid that is coated in a simple protein shell or a cell-membrane-like protein casing, and need to infect host cells to replicate (1). Viruses can cause acute and chronic infections. In acute virus infections, such as a common cold, the virus is typically cleared from the body within a week. However, in some cases, an acute infection is followed by persistence of the virus in the host. Herpes simplex virus is an example of a virus causing a persistent infection, due to ability of the virus to hide in neurons. Often, these types of persistent infections do not cause any symptoms in healthy hosts (2). Chronic infections are a type of persistent infection often caused by an inefficient immune response of the host, leading to long-lasting symptoms. Especially, acute and chronic virus infections have a major general health impact. Annual influenza epidemics, for instance, result in about 3–5 million cases of severe illness and approximately 250,000–500,000 deaths worldwide (3). An example of a chronic infection causing major health impact is Human Immunodeficiency Virus (HIV). In 2012, more than 35 million people were living with an HIV infection and 1.6 million people died from an AIDS-related illness (4). Some persistent virus infections, such as Epstein–Barr virus (EBV) and Human Papillomavirus (HPV) can lead, under certain conditions, to the development of tumors (5, 6). Because viruses have such a major impact on health, strategies to limit or prevent virus infections are of major importance.

**Figure 1:**
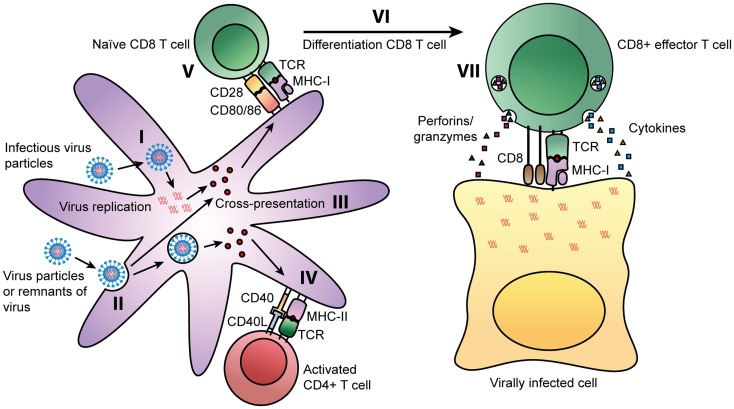
**Routes of presentation of viral peptides on DCs**. Viruses can enter cells by two ways: some viruses can infect cells directly, leading to replication of virus inside the cells. During this process, some of the viral proteins will be degraded into peptide fragments, which will be presented on MHC class I molecules to CD8^+^ T cells (I). APCs, such as DCs can also take up viral particles or remnants of virally infected cells (II). During processing by professional APCs, viral peptides can be presented on MHC class I molecules via the cross-presentation pathway (III). In parallel, these extracellular-derived peptides will be presented on MHC class II molecules. The TCR of virus-specific CD4^+^ T can recognize MHC class II-peptide complexes on professional APCs. Next to the interaction of the MHC class II-peptide complex with the TCR, CD4^+^ T cells can activate DCs by interaction of CD40 with CD40 ligand on the DC (IV). This interaction activates DCs and results in upregulation of maturation markers CD80/CD86. CD80 and CD86 interact with CD28 on naïve CD8^+^ T cells (V). Together with the recognition of the MHC class I-peptide complex by the TCR, CD28 signaling will result in the activation of the CD8^+^ T cell (VI). These activated CD8^+^ T cells will differentiate into effector T cells that can recognize the MHC class I-peptide complex on virally infected cells. Binding of the TCR to the MHC class I-peptide complex leads to activation of the CD8^+^ T cell and the release of cytotoxic granules containing perforins and granzymes, and the production of cytokines such as TNF-α and IFN-γ (VII).

Mammals have developed a refined immune system to cope with all kinds of infections. Especially, the adaptive arm of the immune response is important in limiting and clearing viral infections. The humoral immune response consists of antibodies specific for the virus that can capture and neutralize virus particles before they enter the cell. However, if these antibodies are ineffective, viruses are able to infect host cells and can only be cleared by the cellular arm of the immune response. Once a virus infects a cell, the virus will use the protein-synthesis machinery of the host cell to synthesize its own proteins. During this process, some of the newly synthesized proteins will be degraded into peptide fragments and, if they have sufficient binding affinity, bind to MHC class I molecules. These MHC class I-peptide complexes will then be presented on the cell surface of an infected cell and activated CD8^+^ T cells, specific for the peptide, can recognize the MHC class I-peptide complex and induce apoptosis of the infected cell by releasing cytotoxic granules. Activation of these CD8^+^ T cells occurs in the draining lymph nodes, where antigen-presenting cells (APCs), such as dendritic cells (DCs), and naïve T cells encounter each other. In these lymph nodes, DCs and CD4^+^ T cells provide the co-stimulation necessary for proper activation of CD8^+^ T cells. This process is summarized in Figure 1 and will be further discussed in the next paragraphs.

During the initial phase of a viral infection, there is a significant increase in the number of CD8^+^ T cells. Priming of these naïve T cells will not only occur through the classical pathway via infection of a cell, directly leading to presentation of peptides on MHC class I molecules, but also through cross-presentation. Cross-presentation enables the presentation of viral peptides, taken up from extracellular sources, on MHC class I molecules. Several different cell types have been demonstrated to cross-present antigens *in vivo,* including professional APCs such as macrophages and DCs (7). CD8^+^ T cells, activated either through the classical or cross-presentation pathway, induce apoptosis of virus-infected cells by the release of cytotoxic granules and the production of TNF-α and IFN-γ as depicted in Figure 1. The cytotoxic granules contain perforins, granzymes, and granulysin. Perforins aid in delivering contents of granules into the cytoplasm of the target cell. Granzymes, such as granzyme B, and granulysin activate apoptosis of the target cell. TNF-α can interact with the TNFR-I receptor, which induces apoptosis of infected cells. IFN-γ is an important cytokine in the immune response to various viral infections, since it can induce an antiviral state in uninfected cells and enhance the cytotoxic function of CD8^+^ T cells. By the classical antigen presentation pathway or by the cross-presentation pathway, any form of virus can be presented on MHC class I and MHC class II and thereby stimulate antiviral responses by both CD8^+^ T cells and CD4^+^ T cells, respectively, leading to a broad cellular response to infection (8). After infection, some of these activated T cells will develop into memory T cells. In the event that a secondary infection occurs, these cells can rapidly mature into effector cells and respond to infection.

Antigen-presenting cells that reside at the site of infection, can take up viral particles or remnants of virally infected cells from extracellular sources, and present them on MHC class II molecules. Subsequently, CD4^+^ T cells recognizing peptides in the context of MHC class II will be activated. These activated CD4^+^ T cells are capable of producing a wide range of cytokines and chemokines and can even exert cytotoxic functions themselves. Based on cytokine production, CD4^+^ T cells can be divided into several subsets, the most classical being Th1, Th2, and Tregs. Th1 cells are generally characterized by the production of IFN-γ. Th2 cells, on the other hand, produce mainly IL-4, IL-5, and IL-13 and are important for providing an immune response against helminths by activating eosinophils, basophils, mast cells, and B cells. The third classical subset are the Treg cells, which are characterized by the production of IL-10 and TGF-b, and have mainly regulatory tasks such as dampening effector functions and limiting immunopathology (8, 9). In addition to their effector functions, activated CD4^+^ T cells can provide help to CD8^+^ T cells by CD40-CD40L interaction, which induces up regulation of ligands, such as CD80 and CD86, on DCs. These ligands interact with CD28 on naïve T cells, providing a co-stimulatory signal to activate CD8^+^ T cells (10). The mechanism by which CD4^+^ T cells can provide help to CD8^+^ T cells is shown in Figure 1.

In this review, we will discuss the value of T cell responses in both acute and chronic viral infections and how knowledge of these responses can help in designing effective vaccines.

Currently, antiviral drugs are the main treatment option to combat viral diseases. However, antiviral treatment is associated with side effects and resistance through viral escape. Making use of the hosts own immune defense system by vaccination would be another powerful approach to combat viral diseases. However, many vaccination strategies are based on antibody-mediated protection and are only partially successful. Antibodies can be very efficient in preventing virus infection, but due to the variability of many virus surface proteins, the virus can escape and infect host cells. Once a virus has entered a cell, infection can only be cleared by a cellular response. We will highlight the history of synthetic T cell based vaccines as an important strategy to induce T cell responses and discuss current developments in this field. Then, we will discuss how the design of these vaccines, such as choice of antigen and adjuvant, influences their efficacy. Finally, we will conclude with potential pitfalls and recommendations for the design of effective peptide vaccines against virus infections.

## T Cell Responses in Viral Infections

There are many viruses for which T cells, both CD8^+^ and CD4^+^, have been shown to play a role in protection, such as measles virus, cytomegalovirus (CMV), hepatitis C virus (HCV), and HIV (11–14). In general, an efficient antiviral adaptive response is thought to be of the Th1 type (15). However, many viruses can inhibit this Th1 response by downregulating the production of interferons (16, 17). This type of manipulation of the immune response can greatly influence the outcome of the infection. In infections caused by hepatitis viruses, manipulation of the immune response by the virus can lead to a persistent infection, in which the host is incapable of clearing the virus from the body. In mice, lymphocytic choriomeningitis virus (LCMV) is used as a model to study the role of CD8^+^ and CD4^+^ T cells in both acute and chronic infections. CD8^+^ T cell-deficient mice, which were infected with a LCMV-strain that normally causes acute virus infection, were not able to control infection and developed a persistent infection. In mice depleted of CD4^+^ T cells, infection with murine LCMV led to chronic infection, even in the presence of CD8^+^ T cells. This model shows that in acute infection, CD8^+^ T cells are sufficient to clear infection, but the help of CD4^+^ T cells is required (18).

The importance of T cell responses during acute viral infections in humans can be illustrated by research from Sridhar et al. describing that individuals with higher numbers of pre-existing CD8^+^ T cells specific for conserved CD8 epitopes, developed less severe illness after infection with pandemic H1N1 influenza virus (19). That not only CD8^+^ T cells mediate protection to influenza challenge, has been shown in a unique human challenge study by Wilkinson et al. In this study, healthy volunteers were challenged with influenza A virus, and antibody and T cell responses against influenza before and during infection were monitored. They showed that, in the absence of antibody responses, pre-existing CD4^+^ T cells responding to influenza internal proteins were associated with less severe illness and lower virus shedding. Further characterization of these CD4^+^ T cells showed that these cells had a cytotoxic function (20). These studies describe the importance of both CD8^+^ and CD4^+^ T cells in the immune response against influenza virus.

During chronic viral infections, when the host is not able to clear the virus, the main role of cytotoxic T cells is to limit disease severity and delay disease progression. This is exemplified by studies on HIV infection. Early during infection with HIV, there is a decline in viral replication as measured by the number of HIV RNA copies in plasma samples (21). In the first stages of HIV infection, it has been shown that patients with higher numbers of memory cytotoxic T cells show a much lower viral load in plasma than patients with a lower number of memory cytotoxic T cells, indicating that this decline is mediated by cytotoxic CD4^+^ T cells (22). In addition, cytotoxic CD4^+^ T cells are an immunological predictor of disease outcome. Patients that controlled HIV replication without antiretroviral therapy showed an increased number of CD4^+^ T cells specific for HIV proteins (23). The importance of CD8^+^ T cells in delaying HIV disease progression is shown in studies where a loss of CD8^+^ T cells coincides with disease progression (24, 25). Findings that HIV escape mutations often occur at HLA-binding sites specific for CD8 epitopes, the strong association of certain HLA-alleles with protection from HIV disease progression, the temporal relationship between viral load decline and increase in specific CD8^+^ T cells, and CD8^+^ T cell depletion studies in simian models, underline the importance of CD8^+^ T cell responses (11, 26–29). Knowledge on the mechanism of protection of T cell responses in immunity against viruses can be helpful in designing preventive and therapeutic therapies, such as vaccination.

## History of Peptide Vaccination

Many vaccines against virus infections are based on inducing antibody responses, consequently, these vaccines are often poor inducers of T cell responses (30). Since T cells are important in protection against many viral infections, there is a need for T cell inducing vaccines. By including small protein fragments (peptides), in a vaccine, which can be presented by MHC-molecules to CD4^+^ and CD8^+^ T cells, specific T cell responses can be induced. In Table 1, characteristics of two of the classical preventive vaccines for viral infections, i.e., protein vaccines and live attenuated vaccines, are compared to peptide vaccines. The main advantage of peptide vaccines over classical vaccines is that it is possible to specifically induce T cell responses and that the production process of these vaccines is relatively easy. The first synthetic peptide vaccine able to induce a T cell response in mice was published by Aichele et al. This vaccine contained a 15-mer peptide, derived from the NP protein of LCMV, suspended in incomplete Freund’s adjuvant (IFA) (31). Further experiments showed that these peptide vaccines were able to render a certain amount of protection against challenge with virus (32, 33). These results were promising, but in later studies where mice were vaccinated with 15-mer CTL epitopes derived from adenovirus type 5 early region (Ad5E1) oncogenes in combination with IFA, an enhanced outgrowth of tumors was observed following vaccination (34). In hindsight, this observation might not be that surprising. Only peptides of 20 amino acids or longer will need to be degraded by proteolytic enzymes and are therefore presented exclusively by professional APCs, thereby ensuring sufficient co-stimulation. Shorter peptides can be directly loaded on any MHC molecule, also on non-professional APCs, which may lead to the induction of tolerance. Additional research showed that indeed the problem with the 15-mer adenovirus peptides was induction of tolerance, since they were presented by non-professional APCs lacking appropriate co-stimulation, resulting in suboptimal presentation of the peptide. When mice were vaccinated with peptide-loaded DCs, there was an anti-tumor response and no tolerance induction, showing that presentation of these peptides on professional APCs can be effective without induction of tolerance (35).

**Table 1:** **Comparison of classical protein vaccination, live attenuated vaccination, and peptide vaccination**.

	Classical protein vaccine	Live attenuated vaccine	Peptide vaccine
**Composition**	Inactivated split virion or purified subunit	Attenuated virus, capable of replication	Synthetic, small protein fragments
**Humoral response**	Yes, induces humoral response	Yes, mimics natural infection	Possible, depends on peptides included
**CD4 response**	No	Yes	Yes
**CD8 response**	No	Yes	Yes
**Preexisting response**	Not important	Important, Ab can capture vaccine	Not important
**Adjuvant**	Required for cellular response	Not required	Required
**Production**	Biological	Biological	Synthetic
**Safety**	Risk of contamination with extraneous agents and proteins of the production substrate	Risk of contamination with extraneous agents and proteins of the production substrate	Well controlled and highly pure production process
**Flexibility to match escape variants**	Not easy	Not easy	Easy
**Target conserved components**	No, primarily strain-specific response	To some extent, limited cross-reactivity	Yes, capable of inducing a broad response

*Protein vaccines are a form of inactivated vaccines that consist of purified subunit or subvirion products. Live attenuated vaccines are attenuated viruses, derived from disease-causing virus. These attenuated viruses still replicate in the host, but do not cause disease. Peptide vaccines are completely synthetic vaccines, comprised of small protein fragments*.

## Peptide Length

Thus, the first advantage of peptides of 20 amino acids or longer, which are considered as long peptides, is that they require processing of these peptides by professional APCs, thereby reducing the chance of inducing tolerance by peptide vaccination (36). Furthermore, they may contain multiple epitopes specific for different MHC-molecules. Thereby, broadening the potential response in both the individual and at population level (37). Another advantage of using long peptides is that, next to CD8 epitopes, this type of peptide often contains CD4 epitopes. These CD4 epitopes provide co-stimulation during priming of CD8^+^ T cells and promote memory CD8^+^ T cells (38, 39). One year after the first successful immunization of mice with a free synthetic LCMV peptide, Fayolle et al. described that this 15-mer peptide not only contained a CD8 epitope but also a CD4 epitope (40). This discovery confirms the valuable contribution of co-stimulation in a vaccine. In addition to considering the importance of the length of the peptide, other characteristics are equally or even more important. Therefore, considerations for the choice of antigen will be discussed next.

## Choice of Antigen

Aspects hampering the design of an effective preventive strategy for virus infections are that these viruses have, besides great genetic diversity, also developed multiple mechanisms to evade the host’s immune response (41, 42). A promising approach is to direct the immune response to conserved parts of the virus, which do not allow for mutations. Virtually all viruses contain certain proteins or peptides that are highly conserved. Indeed, for HIV, the Gag protein appears to be a good candidate for use as a T cell vaccine component. The Gag protein is highly conserved, and although it is a late structural protein, Sacha et al. showed in a simian model that CD8^+^ T cells recognize Gag-derived epitopes as early as 2 h post infection. This fast processing and presentation is thought to be necessary for early clearance of the virus (43). In an *ex vivo* study on PBMCs of HIV-infected individuals, vigorous CD8^+^ T cell responses to Gag epitopes were observed and the breadth of the CD8^+^ T cells specific for conserved Gag epitopes inversely correlated with viremia (44). A screening in patients with both acute and chronic HCV infection showed that specific T cell responses were found against conserved parts of the virus. Immunogenic regions were identified within core, NS3 and NS4 proteins (45). Influenza virus also contains good candidate proteins, such as nucleoprotein (NP), which is a major target of T cell responses (46). These studies show that there are T cells available directed toward conserved parts of the virus. Knowledge of these parts can be used in the design of a T cell inducing vaccine.

Since the introduction of sequence analysis tools, it is relatively easy to determine whether a certain peptide sequence is conserved. However, a high level of conservation is not the only requirement for a peptide vaccine to be effective. The peptide will have to be processed by the proteasome and then bind to the MHC molecule. Bio-informatic tools can be helpful to predict, which sequences may be immunogenic for T cells. These tools can predict which sequences will bind to MHC, based on preferred amino acids of peptide anchor binding positions of these molecules. Furthermore, tools are available that predict which sequences will be processed by the proteasome and by TAP (transporter associated with antigen processing) transport (47). Together, these tools provide means of selecting a number of possible conserved T cell epitopes. Schellens et al. showed in PBMCs of HIV-infected individuals that indeed these bio-informatics tools are valuable for predicting novel T cell epitopes (48).

Another important requirement for inducing T cell responses is that there are T cells available that can recognize the peptide. Tan et al. described the importance of the availability of naïve epitope-specific CD8^+^ T cells in the host prior to infection and showed that precursor frequencies are indeed a good predictor for responses observed after infection, since a higher number of epitope-specific CD8^+^ T cells led to an increased T cell response after infection (49). Next to precursor frequencies, binding affinity of peptides to MHC is also a predictor of immunogenicity as has been shown in peripheral blood lymphocytes of acute HBV patients (50). Some groups have shown that it is possible to enhance peptides by increasing binding affinity of the peptide to the MHC molecule (51, 52). These enhanced peptides might induce a T cell response to conserved, but otherwise too low affinity epitopes. Another important consideration when vaccinating with short peptides is HLA-specificity. Since peptides of 8–11 amino acids long bind directly into the MHC class I binding groove the peptide has to match the HLA type of the vaccinated individual. To overcome the need for individualized vaccination, Tan et al. selected short epitopes with the capacity to bind to multiple HLA-alleles. HLA-A2 transgenic mice vaccinated with this multi-HLA peptide vaccine, showed a reduction of virus in the lungs and increased survival following influenza infection, compared with mock vaccinated mice, showing that vaccination with peptides can positively influence disease outcome (53).

Presentation of peptides by APCs greatly depends on the form in which they are offered to APCs. Zhang et al. compared intact proteins and long peptides in the cross-presentation pathway and showed that long peptides traffic to both the endosomes and the cytosol, whereas whole protein was found to traffic only to the endosomal compartments. Therefore, whole proteins could not be processed through the cross-presentation pathway. This difference in processing led to a CD4^+^ T cell restricted response after immunization with protein, while immunization with peptides also led to a CD8^+^ T cell response (54). Rosalia et al. compared whole protein processing to processing of long peptides, both in mouse and in human DCs. Soluble protein antigen ended up mostly in the endolysosomes, while long peptides seemed to be more efficiently internalized by DCs leading to a faster intracellular routing. Therefore, long peptide vaccination ultimately leads to enhanced CD8^+^ T cell activation compared to whole protein (55). In line with these findings, recent research on peptide vaccination is mainly directed to improving antigen presentation of the peptides of choice, by choosing the right form in which the peptides are presented. Rosario et al. used an HIV-synthetic long peptide vaccine to boost HIV-specific T cell responses in a macaque model and showed that boosting with these synthetic long peptides primarily increased the breadth of the CD4^+^ T cell responses (56).

## Features of an Effective Response

To induce an effective response against viral infections, there are several requirements that should be met. One important requirement is that there is a sufficient number of T cells available to kill virus-infected cells. The need for an appropriate magnitude of T cells in order to clear virus was elegantly shown by Thimme et al. in a CD8^+^ T cell depletion study in chimpanzees. Chimpanzees were depleted of CD8^+^ T cells, and subsequently infected with HBV, complete depletion of CD8^+^ T cells in the chimpanzees resulted in the inability to clear virus. When CD8^+^ T cells reappeared in the animal, 98% of viral DNA was eliminated from the liver. However, while the number of CD8^+^ T cells remained suppressed, the animal was not able to clear virus completely. Only when the number of CD8^+^ T cells was able to expand further, the virus was completely eliminated (57). Furthermore, an increased breadth of T cell responses can be beneficial. Analysis of CD8^+^ T cell responses in untreated HIV-infected individuals showed that an increasing breadth of Gag-specific responses is associated with decreased viremia (58). In parallel with these findings, vaccination of mice with a vaccine containing multiple epitopes, were more effective in generating a response to influenza infection than vaccination with single epitopes (49). These findings indicate that a broad response is more effective than a response dedicated to only one peptide. Another advantage of induction of a broad response is that small mutations of the virus will not lead to escape of the virus from the immune response. Next to a broad response, T cell responses of high avidity also contribute to an antiviral response. *Ex vivo* screening of T cell responses in HIV-infected patients showed that controllers reacted to lower antigen concentrations compared to non-controllers, indicating that controllers have T cell responses of higher functional avidity and that this higher avidity is advantageous (59).

A fourth requirement is that an effective antiviral response should be of proper functionality to enable control or clearance of the virus. CD8^+^ T cells are the main cell type that is involved in clearance of viral infections. These cells are characterized by the production of Th1-cytokines such as IFN-γ and by the expression of degranulation marker CD107a (60). CD107a is an indicator of cytotoxic functions such as the production of granzymes and perforins. IFN-γ increases expression of both MHC class I and II molecules and enhances the antigen-presenting function of MHC class I by stimulating loading of peptides onto this molecule. Thereby, IFN-γ can induce the cytotoxic function of CD8^+^ T cells and promote the production of other cytokines such as TNF-α, IL-2, and type I interferons. TNF-α induces apoptosis of virus-infected cells and IL-2 is an important growth factor for T cells. Type I interferons, such as IFN-α and IFN-β, can induce resistance to viral infections in uninfected cells, increase MHC class I expression and antigen presentation and activate both DCs and macrophages (8, 61). Activated macrophages in their turn produce chemokines such as MIP-1β to attract more T cells. Together, these cytokines, chemokines, granzymes, and perforins enable control or clearance of the virus from the host. In HIV infection, a polyfunctional CD8^+^ T cell response is observed in non-progressors, while progressors show a more limited response (62). As reviewed by Seder et al., a polyfunctional response, characterized by production of IFN-γ, TNF-α, and IL-2, was indeed shown to induce more robust T cell proliferation and protection against several viral infections (63).

However, elevated amounts of inflammatory cytokines can also lead to immunopathology as has been shown in H5N1 influenza A virus infection (64). The immune system normally has its own regulatory mechanisms, such as the production of anti-inflammatory cytokines including IL-10 and TGF-β. IL-10 is produced by a wide range of cells, including T cells, macrophages and neutrophils. The main function of IL-10 is to act as a negative feedback loop to suppress the production of IFN-γ and other pro-inflammatory cytokines (65). TGF-β acts by inducing apoptosis of CD8^+^ T cells, which regulates T cell homeostasis and prevents immune inflammation (66). These feedback loops are a way of the immune system to regulate itself, however viral factors can negatively impact this balance as is illustrated in HCV infection. Patients with progressive liver injury showed upregulation of Th1-cytokines IFN-γ and IL-2 and down regulation of the regulatory cytokine IL-10 (67). Another regulatory mechanism is the upregulation of inhibitory receptors such as PD-1, LAG-3, and CTLA-4, which leads to decreased activation potential of T cells and the activation of inhibitory genes in T cells (68). However, upregulation of these receptors has also been shown to be responsible for the exhaustion of T cells and thereby a diminished response in chronic viral infections (69). Summarizing, an effective antiviral response consists of a broad variety of antigen-specific T cells of sufficient magnitude, affinity, and appropriate polyfunctionality. Furthermore, these T cells should be capable of performing cytotoxic functions, but should not induce immunopathology. Such a response greatly depends on the way antigen is presented to the T cells, emphasizing the important role APCs play in antiviral responses.

## Co-Stimulation and Peptide Vaccination

In recent years, multiple strategies were developed to increase the quality of antigen presentation of peptides. One of the strategies, already described above, is the addition of CD4 help. Long peptides often contain CD4 epitopes that can provide co-stimulation for CD8^+^ T cells. However, more general CD4 helper peptides are available. One example is the non-natural pan HLA-DR binding peptide (PADRE), which is engineered by introducing anchor residues for different DR motifs within a polyalanine backbone. This peptide binds with high or intermediate affinity to the most common HLA-DR types, and allows it to activate a wide range of CD4^+^ T cells (70). The addition of PADRE epitopes is used, for example, in Dengue virus and HBV virus vaccine development, showing promising results *in vivo* (71, 72). Another group of universal T helper epitopes are natural tetanus sequences, which are very promiscuous in their capacity to bind to MHC class II, and thereby very efficient in acting as a co-stimulus (73). These universal T helper epitopes can be fused to CD8^+^ T cell epitopes, eliciting good immunogenicity, as shown for CMV by La Rosa et al. (74). However, it remains under debate whether CD4 help should be antigen-specific or is otherwise not able to stimulate proper CD8^+^ T cell responses. A study in which mice were vaccinated with either non-specific CD4 help or antigen-specific CD4 help, showed that memory CD8^+^ T cells can only be efficiently activated by antigen-specific CD4 help, while effector CD8^+^ T cells can be activated by non-specific CD4 help (75).

An important factor in CD4^+^ T cell help in short peptide vaccination appears to be CD40-CD40 ligand interaction (76). Ligation of CD40 to CD40 ligand can trigger the production of high levels of IL-12 by DCs. IL-12 induces Th1-mediated immune responses and inhibits Th2-mediated responses (77). Furthermore, CD40 ligand stimulates up regulation of ICAM-1, CD80, and CD86 molecules on DCs. By these mechanisms, DCs can trigger proliferative responses and IFN-γ production by T cells (78). By adding CD40 ligand as a co-stimulatory molecule, DCs can be activated through CD40 and in their turn, DCs are able to activate CD4^+^ T cells and CD8^+^ T cells (79).

Another way to activate APCs is by targeting their Toll-like receptors (TLRs). TLRs are pathogen recognition receptors (PRR) that recognize molecules shared by pathogens, for example, double stranded RNA in certain viruses. Activation of these TLRs can then lead to the production of inflammatory cytokines. By covalently coupling TLR-activating lipids to the peptide, resulting in so-called lipopeptides, self-adjuvanting peptides are created. These lipopeptides can target the vaccine by activating the TLRs on the required APCs and the peptides can then be internalized and presented on MHC-molecules. Thereby, lipopeptides can signal through the TLRs to induce DC maturation, leading to enhanced antigen presentation. Jackson et al. designed a synthetic vaccine composed of a CD4 T helper epitope, a CD8 target epitope, and the lipid moiety Pam_2_Cys that provided TLR2 targeting, which could induce DC maturation and antibody and CTL responses (80). Chua et al. used the TLR2 agonist Pam_2_Cys to enhance the immunogenicity of their virus-like particles, containing HCV structural proteins. The addition of lipopeptide resulted in increased DC maturation at low doses of the vaccine (81). Indeed, lipopeptide vaccination can induce protective CTL responses, as shown by Day et al. in a mouse influenza virus challenge model (82).

## Adjuvants in Peptide Vaccination

To improve the effectiveness of peptide vaccines, there are several types of adjuvants available, with different effector mechanisms. Some adjuvants induce depot formation; others directly stimulate the immune response through additional signals. In earlier work on peptide vaccination, strong adjuvants were necessary for induction of immunogenicity. A commonly used adjuvant for peptide vaccination is IFA, which was applied in the first peptide vaccine, or the human equivalent Montanide. These water in oil formulations form a depot at the site of injection, leading to “leakage” of antigen into the body (37, 83). Research by den Boer et al. showed that the short Ad5E1 peptide still leaks from the IFA depot at day 200 (84). This depot of antigen and adjuvant can lead to chronic inflammation of the site of injection that may persist for a long time. Harris et al. showed that repeated vaccination can even lead to a site suggestive of a new lymphoid structure, including the association of mature DCs with proliferating T cells in perivascular dermal aggregates (85). However, the risk with such depots is that the peptide might be present for a long time after vaccination, but the adjuvant might not be, allowing presentation of the peptide without the necessary co-stimulation and with the risk of inducing tolerance (86). Furthermore, although effective in therapeutic vaccination, IFA does lead to the formation of lesions on the site of injection, making it less attractive for use in a preventive vaccine (87). Two clinical trials, one with HIV peptides and another with malaria surface proteins mixed in Montanide, have even been terminated because of these severe adverse events (88, 89).

An alternative for water in oil formulations could be the use of vesicular delivery systems. Depending on the nature of the delivery system, they provide the possibility to incorporate immune modulators to direct the immune response, protect against degradation of the peptide, directly target the antigen to the place of interest and, finally, actively transport the antigen across the target membrane. Currently, there are several delivery systems available for peptide vaccination, i.e., liposomes, virosomes, virus-like particles, ISCOMs, and nanoparticles (90). Liposomes consist of a lipid bilayer, in which antigens or other substances can be entrapped in the lumen or the lipid bilayer, depending on traits of the peptide. The lipid bilayer of liposomes can fuse with other bilayers, such as a cell membrane. Thereby, liposomes can deliver antigens to the cytosol of APCs (91). Liposomes, containing a short CD8 lipopeptide in combination with CpG, were able to induce protection in a murine influenza challenge model (92). However, liposomes cannot induce maturation of DCs without addition of an adjuvant and are therefore not sufficient to induce co-stimulation. To address this problem, several groups are developing modified liposomes to increase targeting to DCs by adding targets for C-type lectin receptors such as glycans or mannose, which are typically expressed on DCs (93). Virosomes, or influenza derived virus-like particles, have similar membrane-fusion capacities as live influenza virus, which allows them to actively fuse with cell membranes and thereby deliver antigens directly into the cytosol of APCs leading to cross-presentation of antigenic peptides (94). Furthermore, they have been shown to induce up regulation of maturation markers on bone marrow-derived DCs, in mouse models (95, 96). However, as of now, DC maturation capabilities of virosomes have not been shown in human systems. Thus, liposomes, virosomes, and other delivery systems can successfully be used to deliver antigens to the place of interest. In addition, they can provide the necessary co-stimulation for APCs, either due to their own properties or by adding other adjuvants to the formulation.

## Current Progress in Peptide Vaccination

The first and most successful, peptide-based vaccine that is currently licensed is a therapeutic vaccine against HPV. This vaccine contains long synthetic peptides directed against viral oncoproteins, mixed in Montanide, which induces vaccine-specific CD4^+^ and CD8^+^ T cell responses in all patients (87). Since the success of this therapeutic cancer vaccine, many groups are exploring peptide vaccination for other viral agents. Therapeutic vaccination, with synthetic peptides, of HCV patients not responding to standard treatment, resulted in a decrease in viral RNA as shown in two separate studies. Klade et al. performed a Phase II clinical study in HCV patients with their IC41 vaccine, consisting of five synthetic peptides formulated with a Th1 type adjuvant, poly-I-arginine. All patients that were vaccinated intradermally with TLR7 agonist imiquimod as adjuvant, showed a modest decline in viral titers (97). The study by El-Awady et al., in which HCV patients were vaccinated with a peptide vaccine consisting of three envelope proteins, showed that in two thirds of the patients both antibody and T cell responses were detectable resulting in decreased viral titers (98). However, although these studies provide a proof of concept for peptide vaccination for therapeutic use in HCV infection, the improvements are only minor.

For a preventive peptide vaccine, there are different necessities. First of all, it should target conserved sequences, which could lead to a universal vaccine. Possible target proteins have been identified for viruses such as HIV, HCV, and influenza (44–46). Especially in influenza vaccine development, the threat for a new pandemic to occur has boosted research on the development of such a universal vaccine. The research of Tan et al., in which they make use of lipopeptides directed to conserved components, is one of many examples of strategies that are currently developed and have proven themselves in mouse models but not yet in human systems (53). Other vaccination strategies, currently in development, include the use of virus-like particles in combination with an antibody-inducing influenza protein such as the relatively conserved M2e protein or lipopeptide in combination with liposomes (92, 99, 100). A recent advancement is that there are some peptide-based vaccines against influenza virus infection in Phase I clinical trials, that are able to induce vaccine-specific cellular immunity (101, 102).

## Considerations for Peptide Vaccine Development

When designing peptide-based vaccines, there are several things to take into consideration, such as virus traits, side effects, location of the response, and traits of the host (see Table 2 for an overview). First, the objective of vaccination should be taken into consideration. Vaccines can be largely divided into therapeutic and preventive. Preventive peptide-based vaccines should elicit a robust memory T cell response, since vaccine-induced T cells need to respond rapidly after infection to clear the virus before it causes illness or at least to limit disease burden. In the case of therapeutic vaccination to chronic infections, the response should be vigorous and elongated and a rapid response is of less importance. Both for therapeutic and preventive vaccines, eliciting this response at the required location is of great value. Peptide-based vaccines for respiratory viruses, for example, might be more effective when administered intranasally, since lung resident immune cells might then be primed more easily (103). However, changing the route of administration is not always sufficient and then adjuvants in the form of delivery vehicles might aid in transporting vaccine components to the right location in order to elicit an efficient T cell response.

**Table 2:** **Design of a peptide-based vaccine for preventive or therapeutic use**.

Factor	Preventive	Therapeutic
Route of immunization	Unimportant	Wanted
	Time to develop response	Virus present on certain location
Existing response	Unimportant	Important
	Inducing new response	Boost existing T cell response
Rapid effector response	Wanted	Unimportant
	Preventing or limiting disease	Clearance in the end
Inducing memory	Wanted	Unimportant
	T cells available when infected	Recall response not necessary
Side effects	Unwanted	Unimportant
	Reason to withdraw vaccine	Accepted for certain diseases

*There are several factors to take into consideration when designing peptide-based vaccines, such as location of the response, type of response to be induced, and side effects. The contribution of these factors in the design of preventive versus therapeutic vaccines are summarized in the table*.

Although inducing T cell responses is very important in protection against many pathogens, there are also indications that these T cell responses cause harm. This is illustrated for influenza infection, in which a high number of virus-specific CD4^+^ T cells in patients infected with pandemic influenza A virus from 2009, correlated with more severe illness (104). In the case of HCV infection, a broad and specific T cell response is able to control virus infection (105). However, during chronic viral infection, liver damage occurs, which is assumed to be immune-mediated. In a study by Maini et al., a high number of antigen-specific T cells in the blood did not correlate with the amount of liver damage as measured by alanine transaminase (ALT, indicative of liver damage). In contrast, Feuth et al. show a direct correlation between the number of differentiated CD8^+^ T cells, which contain high perforin levels, and liver fibrosis measured by fibroscan elastography (106). Since a large number of T cells are detected in the liver of patients with liver damage, damage has been proposed to be caused by the recruitment of non-virus-specific T cells (107). Although in humans the mechanism by which immunopathology develops is not clear, it is important to bear in mind that an exaggerated T cell response to infection or vaccination may lead to unwanted immune-mediated damage. Therefore, vaccine-induced T cell responses should be effective against the virus, without eliciting major side effects.

Traits of the host also influence the effectiveness of a vaccine. Therefore, it is important to consider the target group for vaccination. During a human’s lifetime, the immune system will change continuously. Vaccination in early childhood can have a major impact on the immune response in later years as described by Bodewes et al. in which it was shown that annual vaccination with a seasonal inactivated subunit influenza vaccination hampers the development of influenza-specific CD8^+^ T cells (108). To underline this finding, Hoft et al. compared a live attenuated influenza vaccine (LAIV) with a trivalent inactivated influenza vaccine (TIV) in young children, and found that only LAIV induced diverse T cells responses (109). Both studies show that the type of vaccination is of crucial importance both for the induction of T cell responses directly after vaccination and to T cell responses to the pathogen later in life. That age of the target group should be an important factor in the design of a vaccine is further exemplified by a study on influenza vaccination in elderly. In this study, antibody titers did not predict who developed influenza related illness, while T cell responses did (110). This effect is supported by evidence that T cell responses wane in elderly individuals. Several studies have shown that T cells from elderly individuals have a more differentiated phenotype characterized by the lack of CD27 expression and upregulation of CD57. The presence of CD57 on CD8^+^ T cells is associated with decreased proliferation of CD8^+^ T cells. Lack of markers, such as CD28, leads to an increased Th1 skewed response, which may contribute to decreased antibody titers in elderly individuals (111–113). Not only T cell responses wane, but also antibody responses diminish (114). Therefore, age of the target group should be an important consideration for the development of vaccines.

## Prospects for Peptide Vaccination

Taken together, severity of side effects is an important factor in the consideration of vaccine application. The licensed HPV peptide-based vaccine contains Montanide, which is a strong adjuvant causing lesions at the site of infection (87). For the therapeutic HPV vaccine, these side effects were deemed acceptable; however, they were one of the reasons to abort studies with Montanide-containing vaccines for HIV and malaria (88, 89). Consequently, before this peptide-based vaccine concept can be widely implemented, Montanide has to be replaced by another adjuvant. However, to elicit a response to these long overlapping peptides, a strong adjuvant is necessary. Therefore, the challenge is to increase immunogenicity of conserved targets for which T cells are available (43–45). A promising self-adjuvanting approach, which induces a broad response, is using multiple antigenic peptide (MAP). This approach was implemented in HCV patients by El-Awady et al. and was capable of inducing both antibody and T cell responses in two thirds of the patients (98, 115).

The ultimate goal in protection against rapidly mutating viruses such as influenza, is to develop a universal vaccine, protecting against currently circulating influenza strains, but also able to cross-protect against newly emerging strains and thereby preventing future pandemics. These preventive peptide-based vaccines should elicit a robust memory T cell response, since vaccine-induced T cells need to respond rapidly after infection to clear the virus before it causes illness. To induce a pool of both memory CD4^+^ T cells and memory CD8^+^ T cells, efficient priming of naïve T cells is required. Professional APCs need to present the antigen to both CD4^+^ and CD8^+^ T cells. As most vaccines induce T cells via extracellular routing, cross priming is of specific significance since it enables the presentation of extracellular-derived particles on MHC class I molecules. Targeting the more conserved parts of the virus by designing peptide-based vaccines, is a promising concept in the design of these preventive vaccines. Especially in influenza vaccine development, there are several examples of pilot vaccines directed to more conserved parts of the virus that should cross-protect to heterologous viruses. These vaccines often contain both antibody and T cell inducing components (116, 117).

Concluding, in addition to antibody responses, T cell responses are of major importance in limiting and clearing virus infections. Effective therapeutic and preventive vaccines should therefore be able to induce both antibody and T cell responses. Peptide-based vaccines can meet these demands and induce both antibody and T cell responses. Furthermore, because peptides are synthetic, they are safe and relatively easy to produce. Currently, several peptide-based vaccines, for viruses such as EBV, HBV, and influenza virus, are evaluated in clinical trials (101). Hurdles to overcome are choosing the right target epitopes and choosing adjuvants that improve immunogenicity of these epitopes and steer the immune response in the desired direction. Adjuvants for peptide-based vaccines should target antigen to DCs, or other APCs capable of cross-presentation, and provide stimuli to ensure efficient presentation of the antigen. In addition, an overstimulation resulting in immunopathology should be avoided. Providing, these criteria are met, the future of peptide-based vaccines is very promising.

## Conflict of Interest Statement

The authors declare that the research was conducted in the absence of any commercial or financial relationships that could be construed as a potential conflict of interest.

## References

[1] Knipe DM Howley PM Fields Virology. Philadelphia: Lippincott Williams & Wilkins (2013).

[2] Lycke E Hamark B Johansson M Krotochwil A Lycke J Svennerholm B Herpes simplex virus infection of the human sensory neuron. An electron microscopy study. Arch Virol (1988) 101:87–104 10.1007/BF01314654 2843151

[3] WHO. Influenza Factsheet. (2009). Available from: http://www.who.int/mediacentre/factsheets/fs211/en/index.html

[4] UNAIDS. Global Report: UNAIDS Report on the Global AIDS Epidemic 2013. Geneva: UNAIDS (2013).

[5] Epstein MA Achong BG Barr YM Virus particles in cultured lymphoblasts from Burkitt’s lymphoma. Lancet (1964) 1:702–3 10.1016/S0140-6736(64)91524-7 14107961

[6] zur Hausen H Gissmann L Steiner W Dippold W Dreger I Human papilloma viruses and cancer. Bibl Haematol (1975) 569–71 183728 10.1159/000399220

[7] Jung S Unutmaz D Wong P Sano G De Los Santos K Sparwasser T In vivo depletion of CD11c+ dendritic cells abrogates priming of CD8+ T cells by exogenous cell-associated antigens. Immunity (2002) 17:211–20 10.1016/S1074-7613(02)00365-5 12196292 PMC3689299

[8] Murphy KT Walport M Janeway’s Immunobiology. New York: Garland Science (2008).

[9] Zajac AJ Immune response to viruses: cell-mediated immunity. In: Mahy BWJ van Regenmortel MHV, editors. Encyclopedia of Virology. Birmingham, AL: Elsevier (2008). p. 70–7

[10] Zhang S Zhang H Zhao J The role of CD4 T cell help for CD8 CTL activation. Biochem Biophys Res Commun (2009) 384:405–8 10.1016/j.bbrc.2009.04.134 19410556

[11] Schmitz JE Kuroda MJ Santra S Sasseville VG Simon MA Lifton MA Control of viremia in simian immunodeficiency virus infection by CD8+ lymphocytes. Science (1999) 283:857–60 10.1126/science.283.5403.857 9933172

[12] Shoukry NH Grakoui A Houghton M Chien DY Ghrayeb J Reimann KA Memory CD8+ T cells are required for protection from persistent hepatitis C virus infection. J Exp Med (2003) 197:1645–55 10.1084/jem.20030239 12810686 PMC2193956

[13] Snyder CM Buffered memory: a hypothesis for the maintenance of functional, virus-specific CD8(+) T cells during cytomegalovirus infection. Immunol Res (2011) 51:195–204 10.1007/s12026-011-8251-9 22058020

[14] Griffin DE Lin WH Pan CH Measles virus, immune control, and persistence. FEMS Microbiol Rev (2012) 36:649–62 10.1111/j.1574-6976.2012.00330.x 22316382 PMC3319515

[15] Ramshaw IA Ramsay AJ Karupiah G Rolph MS Mahalingam S Ruby JC Cytokines and immunity to viral infections. Immunol Rev (1997) 159:119–35 10.1111/j.1600-065X.1997.tb01011.x 9416507

[16] Guo Z Chen LM Zeng H Gomez JA Plowden J Fujita T NS1 protein of influenza A virus inhibits the function of intracytoplasmic pathogen sensor, RIG-I. Am J Respir Cell Mol Biol (2007) 36:263–9 10.1165/rcmb.2006-0283RC 17053203

[17] Ling Z Tran KC Teng MN Human respiratory syncytial virus nonstructural protein NS2 antagonizes the activation of beta interferon transcription by interacting with RIG-I. J Virol (2009) 83:3734–42 10.1128/JVI.02434-08 19193793 PMC2663251

[18] Matloubian M Concepcion RJ Ahmed R CD4+ T cells are required to sustain CD8+ cytotoxic T-cell responses during chronic viral infection. J Virol (1994) 68:8056–63 7966595 10.1128/jvi.68.12.8056-8063.1994 PMC237269

[19] Sridhar S Begom S Bermingham A Hoschler K Adamson W Carman W Cellular immune correlates of protection against symptomatic pandemic influenza. Nat Med (2013) 19:1305–12 10.1038/nm.3350 24056771

[20] Wilkinson TM Li CK Chui CS Huang AK Perkins M Liebner JC Preexisting influenza-specific CD4+ T cells correlate with disease protection against influenza challenge in humans. Nat Med (2012) 18:274–80 10.1038/nm.2612 22286307

[21] Pantaleo G Fauci AS Immunopathogenesis of HIV infection. Annu Rev Microbiol (1996) 50:825–54 10.1146/annurev.micro.50.1.825 8905100

[22] Musey L Hughes J Schacker T Shea T Corey L McElrath MJ Cytotoxic-T-cell responses, viral load, and disease progression in early human immunodeficiency virus type 1 infection. N Engl J Med (1997) 337:1267–74 10.1056/NEJM199710303371803 9345075

[23] Soghoian DZ Jessen H Flanders M Sierra-Davidson K Cutler S Pertel T HIV-specific cytolytic CD4 T cell responses during acute HIV infection predict disease outcome. Sci Transl Med (2012) 4:123ra125 10.1126/scitranslmed.3003165 22378925 PMC3918726

[24] Klein MR Van Baalen CA Holwerda AM Kerkhof Garde SR Bende RJ Keet IP Kinetics of Gag-specific cytotoxic T lymphocyte responses during the clinical course of HIV-1 infection: a longitudinal analysis of rapid progressors and long-term asymptomatics. J Exp Med (1995) 181:1365–72 10.1084/jem.181.4.1365 7699324 PMC2191947

[25] Rinaldo C Huang XL Fan ZF Ding M Beltz L Logar A High levels of anti-human immunodeficiency virus type 1 (HIV-1) memory cytotoxic T-lymphocyte activity and low viral load are associated with lack of disease in HIV-1-infected long-term nonprogressors. J Virol (1995) 69:5838–42 7637030 10.1128/jvi.69.9.5838-5842.1995 PMC189455

[26] Goulder P Price D Nowak M Rowland-Jones S Phillips R McMichael A Co-evolution of human immunodeficiency virus and cytotoxic T-lymphocyte responses. Immunol Rev (1997) 159:17–29 10.1111/j.1600-065X.1997.tb01004.x 9416500

[27] Jin X Bauer DE Tuttleton SE Lewin S Gettie A Blanchard J Dramatic rise in plasma viremia after CD8(+) T cell depletion in simian immunodeficiency virus-infected macaques. J Exp Med (1999) 189:991–8 10.1084/jem.189.6.991 10075982 PMC2193038

[28] Pereyra F Addo MM Kaufmann DE Liu Y Miura T Rathod A Genetic and immunologic heterogeneity among persons who control HIV infection in the absence of therapy. J Infect Dis (2008) 197:563–71 10.1086/526786 18275276

[29] Bronke C Almeida CA McKinnon E Roberts SG Keane NM Chopra A HIV escape mutations occur preferentially at HLA-binding sites of CD8 T-cell epitopes. AIDS (2013) 27:899–905 10.1097/QAD.0b013e32835e1616 23276808 PMC3818524

[30] Plotkin SA Orenstein WA Offit PA Vaccines. Philadelphia: Elsevier (2013).

[31] Aichele P Hengartner H Zinkernagel RM Schulz M Antiviral cytotoxic T cell response induced by in vivo priming with a free synthetic peptide. J Exp Med (1990) 171:1815–20 10.1084/jem.171.5.1815 1692084 PMC2187909

[32] Kast WM Roux L Curren J Blom HJ Voordouw AC Meloen RH Protection against lethal Sendai virus infection by in vivo priming of virus-specific cytotoxic T lymphocytes with a free synthetic peptide. Proc Natl Acad Sci U S A (1991) 88:2283–7 10.1073/pnas.88.6.2283 1848698 PMC51215

[33] Schulz M Zinkernagel RM Hengartner H Peptide-induced antiviral protection by cytotoxic T cells. Proc Natl Acad Sci U S A (1991) 88:991–3 10.1073/pnas.88.3.991 1992491 PMC50940

[34] Toes RE Offringa R Blom RJ Melief CJ Kast WM Peptide vaccination can lead to enhanced tumor growth through specific T-cell tolerance induction. Proc Natl Acad Sci U S A (1996) 93:7855–60 10.1073/pnas.93.15.7855 8755566 PMC38838

[35] Toes RE Van Der Voort EI Schoenberger SP Drijfhout JW Van Bloois L Storm G Enhancement of tumor outgrowth through CTL tolerization after peptide vaccination is avoided by peptide presentation on dendritic cells. J Immunol (1998) 160:4449–56 9574550

[36] Bijker MS Van Den Eeden SJ Franken KL Melief CJ Van Der Burg SH Offringa R Superior induction of anti-tumor CTL immunity by extended peptide vaccines involves prolonged, DC-focused antigen presentation. Eur J Immunol (2008) 38:1033–42 10.1002/eji.200737995 18350546

[37] Bijker MS Van Den Eeden SJ Franken KL Melief CJ Offringa R Van Der Burg SH CD8+ CTL priming by exact peptide epitopes in incomplete Freund’s adjuvant induces a vanishing CTL response, whereas long peptides induce sustained CTL reactivity. J Immunol (2007) 179:5033–40 17911588 10.4049/jimmunol.179.8.5033

[38] Williams MA Bevan MJ Effector and memory CTL differentiation. Annu Rev Immunol (2007) 25:171–92 10.1146/annurev.immunol.25.022106.141548 17129182

[39] Nakanishi Y Lu B Gerard C Iwasaki A CD8(+) T lymphocyte mobilization to virus-infected tissue requires CD4(+) T-cell help. Nature (2009) 462:510–3 10.1038/nature08511 19898495 PMC2789415

[40] Fayolle C Deriaud E Leclerc C In vivo induction of cytotoxic T cell response by a free synthetic peptide requires CD4+ T cell help. J Immunol (1991) 147:4069–73 1684372

[41] Toussaint NC Maman Y Kohlbacher O Louzoun Y Universal peptide vaccines – optimal peptide vaccine design based on viral sequence conservation. Vaccine (2011) 29:8745–53 10.1016/j.vaccine.2011.07.132 21875632

[42] Liang TJ Current progress in development of hepatitis C virus vaccines. Nat Med (2013) 19:869–78 10.1038/nm.3183 23836237 PMC6263146

[43] Sacha JB Chung C Rakasz EG Spencer SP Jonas AK Bean AT Gag-specific CD8+ T lymphocytes recognize infected cells before AIDS-virus integration and viral protein expression. J Immunol (2007) 178:2746–54 17312117 10.4049/jimmunol.178.5.2746 PMC4520734

[44] Kunwar P Hawkins N Dinges WL Liu Y Gabriel EE Swan DA Superior control of HIV-1 replication by CD8+ T cells targeting conserved epitopes: implications for HIV vaccine design. PLoS One (2013) 8:e64405 10.1371/journal.pone.0064405 23741326 PMC3669284

[45] Lamonaca V Missale G Urbani S Pilli M Boni C Mori C Conserved hepatitis C virus sequences are highly immunogenic for CD4(+) T cells: implications for vaccine development. Hepatology (1999) 30:1088–98 10.1002/hep.510300435 10498664

[46] Grant E Wu C Chan KF Eckle S Bharadwaj M Zou QM Nucleoprotein of influenza A virus is a major target of immunodominant CD8+ T-cell responses. Immunol Cell Biol (2013) 91:184–94 10.1038/icb.2012.78 23399741

[47] Zhao L Zhang M Cong H Advances in the study of HLA-restricted epitope vaccines. Hum Vaccin Immunother (2013) 9:2566–77 23955319 10.4161/hv.26088 PMC4162067

[48] Schellens IM Kesmir C Miedema F Van Baarle D Borghans JA An unanticipated lack of consensus cytotoxic T lymphocyte epitopes in HIV-1 databases: the contribution of prediction programs. AIDS (2008) 22:33–7 10.1097/QAD.0b013e3282f15622 18090389

[49] Tan AC La Gruta NL Zeng W Jackson DC Precursor frequency and competition dictate the HLA-A2-restricted CD8+ T cell responses to influenza A infection and vaccination in HLA-A2.1 transgenic mice. J Immunol (2011) 187:1895–902 10.4049/jimmunol.1100664 21765016

[50] Sette A Vitiello A Reherman B Fowler P Nayersina R Kast WM The relationship between class I binding affinity and immunogenicity of potential cytotoxic T cell epitopes. J Immunol (1994) 153:5586–92 7527444

[51] Berzofsky JA Ahlers JD Belyakov IM Strategies for designing and optimizing new generation vaccines. Nat Rev Immunol (2001) 1:209–19 10.1038/35105075 11905830

[52] Neefjes J Ovaa H A peptide’s perspective on antigen presentation to the immune system. Nat Chem Biol (2013) 9:769–75 10.1038/nchembio.1391 24231618

[53] Tan AC Deliyannis G Bharadwaj M Brown LE Zeng W Jackson DC The design and proof of concept for a CD8(+) T cell-based vaccine inducing cross-subtype protection against influenza A virus. Immunol Cell Biol (2013) 91:96–104 10.1038/icb.2012.54 23146941

[54] Zhang H Hong H Li D Ma S Di Y Stoten A Comparing pooled peptides with intact protein for accessing cross-presentation pathways for protective CD8+ and CD4+ T cells. J Biol Chem (2009) 284:9184–91 10.1074/jbc.M809456200 19193636 PMC2666570

[55] Rosalia RA Quakkelaar ED Redeker A Khan S Camps M Drijfhout JW Dendritic cells process synthetic long peptides better than whole protein, improving antigen presentation and T-cell activation. Eur J Immunol (2013) 43:2554–65 10.1002/eji.201343324 23836147

[56] Rosario M Bridgeman A Quakkelaar ED Quigley MF Hill BJ Knudsen ML Long peptides induce polyfunctional T cells against conserved regions of HIV-1 with superior breadth to single-gene vaccines in macaques. Eur J Immunol (2010) 40:1973–84 10.1002/eji.201040344 20468055

[57] Thimme R Wieland S Steiger C Ghrayeb J Reimann KA Purcell RH CD8(+) T cells mediate viral clearance and disease pathogenesis during acute hepatitis B virus infection. J Virol (2003) 77:68–76 10.1128/JVI.77.1.68-76.2003 12477811 PMC140637

[58] Kiepiela P Ngumbela K Thobakgale C Ramduth D Honeyborne I Moodley E CD8+ T-cell responses to different HIV proteins have discordant associations with viral load. Nat Med (2007) 13:46–53 10.1038/nm1520 17173051

[59] Mothe B Llano A Ibarrondo J Zamarreno J Schiaulini M Miranda C CTL responses of high functional avidity and broad variant cross-reactivity are associated with HIV control. PLoS One (2012) 7:e29717 10.1371/journal.pone.0029717 22238642 PMC3251596

[60] Betts MR Brenchley JM Price DA De Rosa SC Douek DC Roederer M Sensitive and viable identification of antigen-specific CD8+ T cells by a flow cytometric assay for degranulation. J Immunol Methods (2003) 281:65–78 10.1016/S0022-1759(03)00265-5 14580882

[61] Wack A Openshaw P O’Garra A Contribution of cytokines to pathology and protection in virus infection. Curr Opin Virol (2011) 1:184–95 10.1016/j.coviro.2011.05.015 22440716

[62] Betts MR Nason MC West SM De Rosa SC Migueles SA Abraham J HIV nonprogressors preferentially maintain highly functional HIV-specific CD8+ T cells. Blood (2006) 107:4781–9 10.1182/blood-2005-12-4818 16467198 PMC1895811

[63] Seder RA Darrah PA Roederer M T-cell quality in memory and protection: implications for vaccine design. Nat Rev Immunol (2008) 8:247–58 10.1038/nri2274 18323851

[64] de Jong MD Simmons CP Thanh TT Hien VM Smith GJ Chau TN Fatal outcome of human influenza A (H5N1) is associated with high viral load and hypercytokinemia. Nat Med (2006) 12:1203–7 10.1038/nm1477 16964257 PMC4333202

[65] Saraiva M O’Garra A The regulation of IL-10 production by immune cells. Nat Rev Immunol (2010) 10:170–81 10.1038/nri2711 20154735

[66] Gorelik L Flavell RA Abrogation of TGFbeta signaling in T cells leads to spontaneous T cell differentiation and autoimmune disease. Immunity (2000) 12:171–81 10.1016/S1074-7613(00)80170-3 10714683

[67] Napoli J Bishop GA McGuinness PH Painter DM McCaughan GW Progressive liver injury in chronic hepatitis C infection correlates with increased intrahepatic expression of Th1-associated cytokines. Hepatology (1996) 24:759–65 10.1002/hep.510240402 8855173

[68] Odorizzi PM Wherry EJ Inhibitory receptors on lymphocytes: insights from infections. J Immunol (2012) 188:2957–65 10.4049/jimmunol.1100038 22442493 PMC3320038

[69] Blackburn SD Shin H Haining WN Zou T Workman CJ Polley A Coregulation of CD8+ T cell exhaustion by multiple inhibitory receptors during chronic viral infection. Nat Immunol (2009) 10:29–37 10.1038/ni.1679 19043418 PMC2605166

[70] Alexander J Sidney J Southwood S Ruppert J Oseroff C Maewal A Development of high potency universal DR-restricted helper epitopes by modification of high affinity DR-blocking peptides. Immunity (1994) 1:751–61 10.1016/S1074-7613(94)80017-0 7895164

[71] Li S Peng L Zhao W Zhong H Zhang F Yan Z Synthetic peptides containing B- and T-cell epitope of dengue virus-2 E domain III provoked B- and T-cell responses. Vaccine (2011) 29:3695–702 10.1016/j.vaccine.2011.03.002 21419774

[72] Wang H Su X Zhang P Liang J Wei H Wan M Recombinant heat shock protein 65 carrying PADRE and HBV epitopes activates dendritic cells and elicits HBV-specific CTL responses. Vaccine (2011) 29:2328–35 10.1016/j.vaccine.2010.12.124 21251902

[73] Panina-Bordignon P Tan A Termijtelen A Demotz S Corradin G Lanzavecchia A Universally immunogenic T cell epitopes: promiscuous binding to human MHC class II and promiscuous recognition by T cells. Eur J Immunol (1989) 19:2237–42 10.1002/eji.1830191209 2481588

[74] La Rosa C Longmate J Lacey SF Kaltcheva T Sharan R Marsano D Clinical evaluation of safety and immunogenicity of PADRE-cytomegalovirus (CMV) and tetanus-CMV fusion peptide vaccines with or without PF03512676 adjuvant. J Infect Dis (2012) 205:1294–304 10.1093/infdis/jis107 22402037 PMC3308906

[75] Gao FG Khammanivong V Liu WJ Leggatt GR Frazer IH Fernando GJ Antigen-specific CD4+ T-cell help is required to activate a memory CD8+ T cell to a fully functional tumor killer cell. Cancer Res (2002) 62:6438–41 12438231

[76] Schoenberger SP Toes RE Van Der Voort EI Offringa R Melief CJ T-cell help for cytotoxic T lymphocytes is mediated by CD40-CD40L interactions. Nature (1998) 393:480–3 10.1038/31002 9624005

[77] Trinchieri G Interleukin-12 and its role in the generation of TH1 cells. Immunol Today (1993) 14:335–8 10.1016/0167-5699(93)90230-I 8103338

[78] Cella M Scheidegger D Palmer-Lehmann K Lane P Lanzavecchia A Alber G Ligation of CD40 on dendritic cells triggers production of high levels of interleukin-12 and enhances T cell stimulatory capacity: T-T help via APC activation. J Exp Med (1996) 184:747–52 10.1084/jem.184.2.747 8760829 PMC2192696

[79] Melchers M Matthews K De Vries RP Eggink D Van Montfort T Bontjer I A stabilized HIV-1 envelope glycoprotein trimer fused to CD40 ligand targets and activates dendritic cells. Retrovirology (2011) 8:48 10.1186/1742-4690-8-48 21689404 PMC3141652

[80] Jackson DC Lau YF Le T Suhrbier A Deliyannis G Cheers C A totally synthetic vaccine of generic structure that targets Toll-like receptor 2 on dendritic cells and promotes antibody or cytotoxic T cell responses. Proc Natl Acad Sci U S A (2004) 101:15440–5 10.1073/pnas.0406740101 15489266 PMC523460

[81] Chua BY Johnson D Tan A Earnest-Silveira L Sekiya T Chin R Hepatitis C VLPs delivered to dendritic cells by a TLR2 targeting lipopeptide results in enhanced antibody and cell-mediated responses. PLoS One (2012) 7:e47492 10.1371/journal.pone.0047492 23091628 PMC3472981

[82] Day EB Zeng W Doherty PC Jackson DC Kedzierska K Turner SJ The context of epitope presentation can influence functional quality of recalled influenza A virus-specific memory CD8+ T cells. J Immunol (2007) 179:2187–94 17675478 10.4049/jimmunol.179.4.2187

[83] Salerno EP Shea SM Olson WC Petroni GR Smolkin ME McSkimming C Activation, dysfunction and retention of T cells in vaccine sites after injection of incomplete Freund’s adjuvant, with or without peptide. Cancer Immunol Immunother (2013) 62:1149–59 10.1007/s00262-013-1435-5 23657629 PMC3813823

[84] den Boer AT Diehl L Van Mierlo GJ Van Der Voort EI Fransen MF Krimpenfort P Longevity of antigen presentation and activation status of APC are decisive factors in the balance between CTL immunity versus tolerance. J Immunol (2001) 167:2522–8 11509591 10.4049/jimmunol.167.5.2522

[85] Harris RC Chianese-Bullock KA Petroni GR Schaefer JT Brill LB II Molhoek KR The vaccine-site microenvironment induced by injection of incomplete Freund’s adjuvant, with or without melanoma peptides. J Immunother (2012) 35:78–88 10.1097/CJI.0b013e31823731a4 22130163 PMC3282210

[86] Welters MJ Bijker MS Van Den Eeden SJ Franken KL Melief CJ Offringa R Multiple CD4 and CD8 T-cell activation parameters predict vaccine efficacy in vivo mediated by individual DC-activating agonists. Vaccine (2007) 25:1379–89 10.1016/j.vaccine.2006.10.049 17123670

[87] Kenter GG Welters MJ Valentijn AR Lowik MJ Berends-Van Der Meer DM Vloon AP Vaccination against HPV-16 oncoproteins for vulvar intraepithelial neoplasia. N Engl J Med (2009) 361:1838–47 10.1056/NEJMoa0810097 19890126

[88] Wu Y Ellis RD Shaffer D Fontes E Malkin EM Mahanty S Phase 1 trial of malaria transmission blocking vaccine candidates Pfs25 and Pvs25 formulated with montanide ISA 51. PLoS One (2008) 3:e2636 10.1371/journal.pone.0002636 18612426 PMC2440546

[89] Graham BS McElrath MJ Keefer MC Rybczyk K Berger D Weinhold KJ Immunization with cocktail of HIV-derived peptides in montanide ISA-51 is immunogenic, but causes sterile abscesses and unacceptable reactogenicity. PLoS One (2010) 5:e11995 10.1371/journal.pone.0011995 20706632 PMC2919382

[90] Foged C Hansen J Agger EM License to kill: formulation requirements for optimal priming of CD8(+) CTL responses with particulate vaccine delivery systems. Eur J Pharm Sci (2012) 45:482–91 10.1016/j.ejps.2011.08.016 21888971

[91] Gregoriadis G Ryman BE Liposomes as carriers of enzymes or drugs: a new approach to the treatment of storage diseases. Biochem J (1971) 124:58 10.1042/bj1240058p PMC1177319 5130994

[92] Matsui M Kohyama S Suda T Yokoyama S Mori M Kobayashi A A CTL-based liposomal vaccine capable of inducing protection against heterosubtypic influenza viruses in HLA-A*0201 transgenic mice. Biochem Biophys Res Commun (2010) 391:1494–9 10.1016/j.bbrc.2009.12.100 20060099

[93] Unger WW Van Beelen AJ Bruijns SC Joshi M Fehres CM Van Bloois L Glycan-modified liposomes boost CD4+ and CD8+ T-cell responses by targeting DC-SIGN on dendritic cells. J Control Release (2012) 160:88–95 10.1016/j.jconrel.2012.02.007 22366522

[94] Bungener L Idema J Ter Veer W Huckriede A Daemen T Wilschut J Virosomes in vaccine development: induction of cytotoxic T lymphocyte activity with virosome-encapsulated protein antigens. J Liposome Res (2002) 12:155–63 10.1081/LPR-120004789 12604050

[95] Huckriede A Bungener L Stegmann T Daemen T Medema J Palache AM The virosome concept for influenza vaccines. Vaccine (2005) 23(Suppl 1):S26–38 10.1016/j.vaccine.2005.04.026 16026906

[96] Kamphuis T Meijerhof T Stegmann T Lederhofer J Wilschut J De Haan A Immunogenicity and protective capacity of a virosomal respiratory syncytial virus vaccine adjuvanted with monophosphoryl lipid A in mice. PLoS One (2012) 7:e36812 10.1371/journal.pone.0036812 22590614 PMC3348902

[97] Klade CS Schuller E Boehm T Von Gabain A Manns MP Sustained viral load reduction in treatment-naive HCV genotype 1 infected patients after therapeutic peptide vaccination. Vaccine (2012) 30:2943–50 10.1016/j.vaccine.2012.02.070 22401867

[98] El-Awady MK El Gendy M Waked I Tabll AA El Abd Y Bader El Din N Immunogenicity and safety of HCV E1E2 peptide vaccine in chronically HCV-infected patients who did not respond to interferon based therapy. Vaccine (2013). 10.1016/j.vaccine.2013.07.074 23962537

[99] Ninomiya A Ogasawara K Kajino K Takada A Kida H Intranasal administration of a synthetic peptide vaccine encapsulated in liposome together with an anti-CD40 antibody induces protective immunity against influenza A virus in mice. Vaccine (2002) 20:3123–9 10.1016/S0264-410X(02)00261-X 12163263

[100] Gao X Wang W Li Y Zhang S Duan Y Xing L Enhanced Influenza VLP vaccines comprising matrix-2 ectodomain and nucleoprotein epitopes protects mice from lethal challenge. Antiviral Res (2013) 98:4–11 10.1016/j.antiviral.2013.01.010 23416215

[101] NIAID. The Jordan Report: Accelerated Development of Vaccines. Appendix A: Status of Vaccine Research and Development. National Institute of Allergy and Infectious Diseases (2012). p. 153–78

[102] Pleguezuelos O Robinson S Stoloff GA Caparros-Wanderley W Synthetic Influenza vaccine (FLU-v) stimulates cell mediated immunity in a double-blind, randomised, placebo-controlled Phase I trial. Vaccine (2012) 30:4655–60 10.1016/j.vaccine.2012.04.089 22575166

[103] Rose MA Zielen S Baumann U Mucosal immunity and nasal influenza vaccination. Expert Rev Vaccines (2012) 11:595–607 10.1586/erv.12.31 22827245

[104] Zhao Y Zhang YH Denney L Young D Powell TJ Peng YC High levels of virus-specific CD4+ T cells predict severe pandemic influenza A virus infection. Am J Respir Crit Care Med (2012) 186:1292–7 10.1164/rccm.201207-1245OC 23087026

[105] Rehermann B Fowler P Sidney J Person J Redeker A Brown M The cytotoxic T lymphocyte response to multiple hepatitis B virus polymerase epitopes during and after acute viral hepatitis. J Exp Med (1995) 181:1047–58 10.1084/jem.181.3.1047 7532675 PMC2191941

[106] Feuth T Arends JE Lieveld FI Mundt MW Hoepelman AI Siersema PD Impact of transient elastography on clinical decision-making in patients with chronic viral hepatitis. Scand J Gastroenterol (2013) 48:1074–81 10.3109/00365521.2013.819441 23886398

[107] Maini MK Boni C Lee CK Larrubia JR Reignat S Ogg GS The role of virus-specific CD8(+) cells in liver damage and viral control during persistent hepatitis B virus infection. J Exp Med (2000) 191:1269–80 10.1084/jem.191.8.1269 10770795 PMC2193131

[108] Bodewes R Fraaij PL Geelhoed-Mieras MM Van Baalen CA Tiddens HA Van Rossum AM Annual vaccination against influenza virus hampers development of virus-specific CD8(+) T cell immunity in children. J Virol (2011) 85:11995–2000 10.1128/JVI.05213-11 21880755 PMC3209321

[109] Hoft DF Babusis E Worku S Spencer CT Lottenbach K Truscott SM Live and inactivated influenza vaccines induce similar humoral responses, but only live vaccines induce diverse T-cell responses in young children. J Infect Dis (2011) 204:845–53 10.1093/infdis/jir436 21846636 PMC3156924

[110] McElhaney JE Xie D Hager WD Barry MB Wang Y Kleppinger A T cell responses are better correlates of vaccine protection in the elderly. J Immunol (2006) 176:6333–9 16670345 10.4049/jimmunol.176.10.6333

[111] Saurwein-Teissl M Lung TL Marx F Gschosser C Asch E Blasko I Lack of antibody production following immunization in old age: association with CD8(+)CD28(−) T cell clonal expansions and an imbalance in the production of Th1 and Th2 cytokines. J Immunol (2002) 168:5893–9 12023394 10.4049/jimmunol.168.11.5893

[112] Brenchley JM Karandikar NJ Betts MR Ambrozak DR Hill BJ Crotty LE Expression of CD57 defines replicative senescence and antigen-induced apoptotic death of CD8+ T cells. Blood (2003) 101:2711–20 10.1182/blood-2002-07-2103 12433688

[113] Chen WH Kozlovsky BF Effros RB Grubeck-Loebenstein B Edelman R Sztein MB Vaccination in the elderly: an immunological perspective. Trends Immunol (2009) 30:351–9 10.1016/j.it.2009.05.002 19540808 PMC3739436

[114] Blomberg BB Frasca D Quantity, not quality, of antibody response decreased in the elderly. J Clin Invest (2011) 121:2981–3 10.1172/JCI58406 21785210 PMC3148749

[115] El-Awady MK Tabll AA Yousif H El-Abd Y Reda M Khalil SB Murine neutralizing antibody response and toxicity to synthetic peptides derived from E1 and E2 proteins of hepatitis C virus. Vaccine (2010) 28:8338–44 10.1016/j.vaccine.2009.11.059 19995542

[116] Testa JS Shetty V Hafner J Nickens Z Kamal S Sinnathamby G MHC class I-presented T cell epitopes identified by immunoproteomics analysis are targets for a cross reactive influenza-specific T cell response. PLoS One (2012) 7:e48484 10.1371/journal.pone.0048484 23144892 PMC3492461

[117] Zhou C Zhou L Chen YH Immunization with high epitope density of M2e derived from 2009 pandemic H1N1 elicits protective immunity in mice. Vaccine (2012) 30:3463–9 10.1016/j.vaccine.2012.03.021 22446634

